# In Situ Inclusion Detection and Material Characterization in an Electron Beam Powder Bed Fusion Process Using Electron Optical Imaging

**DOI:** 10.3390/ma16124220

**Published:** 2023-06-07

**Authors:** Martin Gardfjell, Marcel Reith, Martin Franke, Carolin Körner

**Affiliations:** 1 Neue Materialien Fürth GmbH, 90762 Fürth, Germany; 2 Materials Science and Engineering for Metals (WTM), Martensstr. 5., 91058 Erlangen, Germany

**Keywords:** Additive Manufacturing, Electron Beam Powder Bed Fusion, Process Monitoring, Backscattered Electron Detection, Electron Optical Imaging, Powder Contamination, Inclusion Detection

## Abstract

Electron Beam Powder Bed Fusion (PBF-EB) is an Additive Manufacturing (AM) method that utilizes an electron beam to melt and consolidate metal powder. The beam, combined with a backscattered electron detector, enables advanced process monitoring, a method termed Electron Optical Imaging (ELO). ELO is already known to provide great topographical information, but its capabilities regarding material contrast are less studied. In this article the extents of material contrast using ELO are investigated, focusing mainly on identifying powder contamination. It will be shown that an ELO detector is capable of distinguishing a single 100 μm foreign powder particle, during an PBF-EB process, if the backscattering coefficient of the inclusion is sufficiently higher than its surroundings. Additionally, it is investigated how the material contrast can be used for material characterization. A mathematical framework is provided to describe the relationship between the signal intensity in the detector and the effective atomic number $Z^{\mathrm{eff}}$ of the imaged alloy. The approach is verified with empirical data from twelve different materials, demonstrating that the effective atomic number of an alloy can be predicted to within one atomic number from its ELO intensity.

## 1. Introduction

The world is entering the Fourth Industrial Revolution, where Additive Manufacturing (AM), together with other emerging technologies, is fundamentally changing global manufacturing. Electron Beam Powder Bed Fusion (PBF-EB) is an AM method that utilizes an electron beam to melt and consolidate metal powder in layers 50–100 µm thick [1]. The combination of an electron beam and layer-by-layer consolidation allows for advanced in situ process monitoring using Backscattered Electrons (BSE). This method of process monitoring is termed Electron Optical imaging (ELO). During each layer of a PBF-EB process, an ELO image can be taken directly after melting, providing instant high-resolution feedback to the operator.

ELO is based on the same phenomenology as traditional Scanning Electron Microscopy (SEM) [2]. It differentiates itself from SEM due to how it is adapted to the large scale, high energy and high temperature conditions of a PBF-EB process, as well as the challenges of metal vapor condensation and high energy x-rays [3]. The first implementation of ELO was introduced over 50 years ago for weld seam detection in the field of electron beam welding [4]. The first academic implementation of ELO imaging used for an PBF-EB system was introduced by C. Arnold in 2018 in cooperation with pro-beam GmbH & Co. KGaA (Gilching, Germany) [2]. The technology has since 2018 also been implemented commercially by Freemelt AB (Mölndal, Sweden).

ELO imaging has been shown to provide excellent topographical information [2,3,5,6]. Applications include: developing process windows for new materials [7], developing part-specific scan strategies for complex geometries [8,9] and process monitoring for defect detection [10]. Less studied are the capabilities of using ELO for material contrast. Understanding and quantifying material contrast in an ELO image opens the possibility for new applications within process monitoring: inclusion detection and material characterization.

PBF-EB is still a young field of manufacturing compared to traditional manufacturing methods such as casting and machining. PBF-EB, and other AM methods, struggle to prove their viability in the general manufacturing community, mainly due to uncertainty in quality and high cost. Process monitoring is essential to target these issues. Traditional manufacturing methods rely on expensive and/or destructive methods of quality assurance, such as X-ray Computed Tomography (XCT). With ELO, PBF-EB is capable of increasing quality assurance by closely monitoring the quality of every single layer at an almost negligible cost. ELO, and other methods of process monitoring, are therefore key to making PBF-EB commercially viable for a broader range of industrial applications [11].

In this article it will be demonstrated that a single foreign powder particle is detectable on a novel PBF-EB system, named *HELIOS*, equipped with a single-detector ELO system. The study also aims to expand the general knowledge of material contrast in ELO by providing a mathematical framework describing the relationship between the effective atomic number $Z^{\mathrm{eff}}$ of an imaged alloy and the ELO signal intensity measured in the detector. The approach is calibrated and verified using empirical data measured from nine pure elements and three common alloys. Using this framework, the article investigates the potential of not only detecting an inclusion, but also characterizing the unknown material. The underlying physical phenomena required to understand material contrast in ELO will be presented in sufficient detail and its impact on the viability of PBF-EB industrially will be discussed.

## 2. BSE Detection Theory

An ELO picture is an intensity map achieved by recording the number of electrons absorbed in the detector while scanning the surface with an electron beam. Each pixel corresponds to a position on the build surface and its intensity is proportional to the number of electrons detected with the beam at that position. In order to interpret the contrast in an ELO image, the fundamental principles of electron diffusion and scattering have to be understood. The complexity of an arbitrary backscattering process is too large to model in its entirety and therefore certain simplifications have to be made in order to make the phenomenological model useful in practice. In this section a series of theoretical explanations will be given in order to divide the complexity into individual factors.

### 2.1. Beam Characteristics

An electron beam is created by emitting and accelerating electrons from a heated filament in the top of the column, whereafter the beam is focused onto the surface of the build area using a magnetic lens system. Ideally all the electrons would have the same energy while traveling straight down the optical axis. In practice however, the energy of the electrons, their spatial position within the beam and their radial velocity will vary [12]. The magnitude of the variation depends on the precision of the machine, and the variation should be anticipated to increase with higher beam deflection. In ELO imaging, the resolution of a single pixel is mainly limited by the beam quality. The beam quality is usually defined only through a beam diameter, which is a crude, but effective, way of condensing the beam quality into a single metric. A beam diameter can be defined in a multitude of ways. In this article the 4σ-diameter (D4σ) definition will be used, in which the diameter is defined as four times the standard deviation σ of the spatial distribution of the electrons [13]. Assuming a Gaussian distribution, 86.47% of the electrons will hit the surface within the D4σ area. Generalized, this percentage is $1-e^{-\frac{r^{2}}{2\sigma^{2}}}$ for any radius *r*.

### 2.2. Electron–Material Interaction

**Figure 1:**
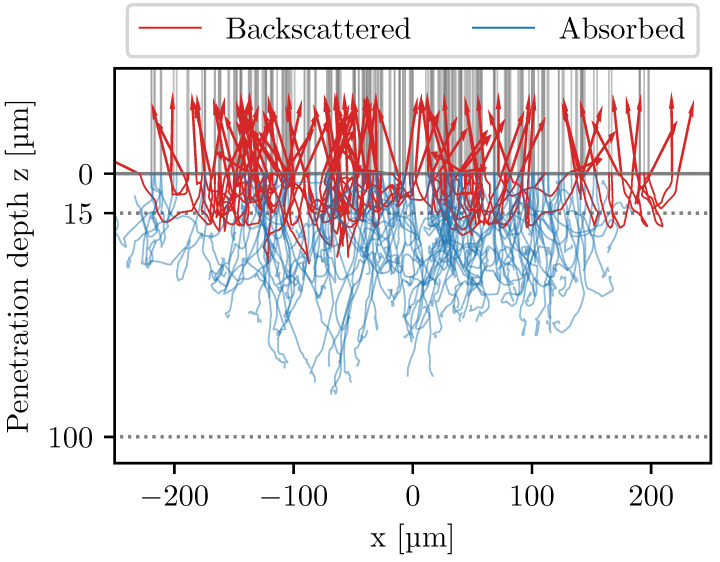
Monte Carlo simulation of the electron–material interaction, computed with the CASINO software [15]. The PEs (gray) travel downwards and penetrate the surface at z=0. The electrons scatter until they are absorbed (blue) or scatter back through the surface, becoming BSEs (red). The simulation was made with TNM and D4σ=400 μm. The horizontal dotted lines indicate the 15.0 μm probing depth and 100 μm layer thickness.

The Primary Electrons (PE) that enter the surface will elastically and inelastically scatter once they start interacting with the atoms of the bulk material. The scattering, i.e., the interaction between an incident electron and a crystal lattice atom, can be explained using the Mott cross-section [14]. There is no feasible way to analytically describe the scattering of an entire electron beam within an arbitrary bulk material lattice structure. Therefore, Monte Carlo simulations, such as the CASINO software [15], are used to describe the macroscopic effects of the scattering, supported by empirical values from experimental studies. One such simulation can be seen in Figure 1. The main macroscopic variable of interest is the backscattering coefficient η, which is defined as the ratio between the number of PEs ($N_{PE}$) that enter surface and the number of BSEs ($N_{BSE}$) that exit the surface, i.e., the ratio between the currents $I_{PE}$ and $I_{BSE}$. The opposite is the absorption coefficient $\eta_{A}=1-\eta$.

(1) $$\eta =\frac{N_{BSE}}{N_{PE}}=\frac{I_{BSE}}{I_{PE}}$$

For backscattering to occur, the velocity of the PEs has to be more or less reversed, which requires large angle inelastic scattering. This occurs primarily when the PEs interact with the nucleus of the atom. The Coulomb force that dictates this interaction will be proportional to the atomic number Z of the atom and therefore the probability of backscattering, i.e., the backscattering coefficient η, will be highly dependent on the material composition. A simple but robust relationship for η(Z) was proposed by F. Arnal in 1969 [16,17].

(2) $$\eta \left(Z\right)=2^{\frac{-9}{\sqrt{Z}}}$$

The likelihood of backscattering decreases the deeper an electron penetrates the specimen. A relationship for the exit depth $T=2.8E^{1.54}$ has been observed in thin film experiments, where *E* is the electron energy in keV [17,18]. The exit depth T has the peculiar unit μg/cm${}^{2}$, and the actual probing depth can be calculated by dividing T with the density ρ of the material. As an example, T=6285 μg/cm${}^{2}$, for 150 keV, and for TNM (ρ=4.2 g/cm${}^{3}$) this results in a probing depth of 15.0 μm, which coincides with the simulation seen in Figure 1.

### 2.3. Backscattered Electron Detection

The electrons that escape the surface scatter in all directions. Ideally a hemispherical detector covering all scattering angles should be used in order to accurately measure the backscattering coefficient. The measured current should for such a detector be equal to $I_{BSE}\;\cdot \;\eta_{A}$, where $\eta_{A}$ is the absorption coefficient of the detector. For practical reasons, a hemispherical detector is not feasible in an AM machine. The geometry of the detector and chamber will therefore also affect the signal intensity. The ELO detector in *HELIOS* is geometrically an annulus with a 40 mm inner radius and 60 mm outer radius. Practically it is an aluminum plate, located 600 mm above the build surface, with a circular opening allowing the beam to pass through. It is made from aluminum to increase electron absorption. It is shielded by a stainless-steel plate with a slightly larger hole, exposing only the thin annulus. The solid angle $\Omega_{|}$ of the ELO detector (calculated from the center of the build area) is only 0.275% of the total 2π steradian hemisphere. Another identical detector is placed directly behind the ELO detector as a reference, measuring the electrical fluctuations in the machine. Its signal is subtracted from the ELO detectors before it is amplified to reduce noise. In HELIOS the BSE current is transformed into a voltage using a transimpedence amplifier circuit, which adds the influence of the resistance *R* of the amplifier (∼10 kΩ for *HELIOS*) and the signal amplification factor *A*. Equation (3), inspired by Equation (2), is introduced to model the relationship $\bar{U}\left(Z\right)$.

(3) $$\bar{U}\left(Z\right)=I_{PE}\;\cdot \;k\;\cdot \;\left(2^{\frac{-l}{\sqrt{Z}}}+m\right)$$

The constants *k*, *l* and *m* are introduced to account for the various influences that differentiate the ELO detector from an ideal backscattering detector. Physically, *k* can be interpreted as a scaling factor containing $\Omega_{|}$, *R*, *A* and $\eta_{A}$. Equation (2) is suitable for the larger atomic numbers of metals, but not for smaller ones close to zero. Introducing *m* relaxes the function, removing the criteria that it has to pass through the origin. *m* also represents all non-BSE electrons that are absorbed by the detector. The inverse relationship $\bar{Z}\left(U\right)$ is helpful to define as well. Note that the $\bar{\mathrm{bar}}$ notation will be used to distinguish estimated voltages (and atomic numbers) from measured (actual) values.

(4) $$\bar{Z}\left(U\right)={\left(\frac{-l\;\cdot \;\ln2}{\ln\left(\frac{U}{I_{PE}\;\cdot \;k}-m\right)}\right)}^{2}$$

The values of *k*, *l* and *m* in Equation (3) are determined experimentally for *HELIOS* in this study from n=12 different materials. To estimate the mean square error of the fitting, $\epsilon_{U}$ and $\epsilon_{Z}$ are defined:

(5) $$\epsilon_{U}=\sqrt{\frac{1}{n}\sum_{i=1}^{n}{\left[\bar{U}\left(Z_{i}\right)-U_{i}\right]}^{2}}\;\epsilon_{Z}=\sqrt{\frac{1}{n}\sum_{i=1}^{n}{\left[\bar{Z}\left(U_{i}\right)-Z_{i}\right]}^{2}}$$

### 2.4. Effective Atomic Number of Alloys

An alloy Γ does not have an atomic number $Z_{\Gamma }$, but it will have a backscattering coefficient $\eta_{\Gamma }$, which can be estimated as a linear combination of the backscattering coefficients $\eta_{i}$ of the individual alloying elements i∈Γ, with mass concentrations $c_{i}$ as coefficients, as shown by Herrman et al. [19]. This can be interpreted as Γ having an effective atomic number $Z_{\Gamma }^{\mathrm{eff}}$.

(6) $$\eta_{\Gamma }^{\mathrm{eff}}=\eta \left(Z_{\Gamma }^{\mathrm{eff}}\right)=\sum_{i\in \Gamma }c_{i}\eta \left(Z_{i}\right)$$

When working with an ELO detector, the actual backscattering coefficients are not required to be known. Substituting η with *U* into Equation (6) simplifies the relationship between $\bar{Z_{\Gamma }^{\mathrm{eff}}}$ and the measured voltages $U_{i}$.

(7) $${\bar{Z}}_{\Gamma }^{\mathrm{eff}}=\bar{Z}\left(U_{\Gamma }^{\mathrm{eff}}\right)\;U_{\Gamma }^{\mathrm{eff}}=\sum_{i\in \Gamma }c_{i}U_{i}$$

It should be mentioned that the relationship is not *injective*, i.e., different alloys can have the same $Z^{\mathrm{eff}}$. Therefore, the material contrast of an ELO image can never unambiguously determine the elemental composition of the specimen. It does however greatly reduce the number of options. In a scenario where a factory works with a selection of different powders, the ELO could potentially determine if a contamination originates from their own powder handling or not.

### 2.5. STSA-Contrast

**Figure 2:**
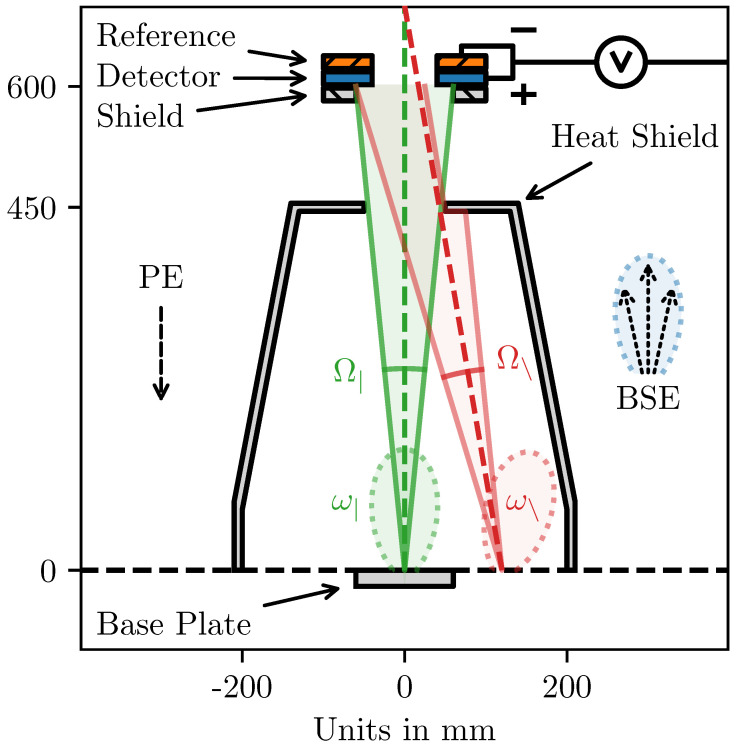
A 2D cross-section of the chamber and detector geometry of *HELIOS* presenting a simplified schematic of the phenomenons that give rise to STSA-contrast. The PEs travel down the column, through the center of the ELO detector, hitting the build surface at z=0. The BSEs scatter with the angular distribution ω, but only those which scatter within the solid angle Ω will hit the detector, if they manage to pass through the opening in the heat shield at z=450 mm. The centered beam (green) and the highly deflected beam (red) illustrate how increased deflection decreases signal intensity, giving rise to the STSA-contrast. All dimensions are to scale, except the angular distributions ω, which is much more complicated than the simple distributions visualized here. Several other effects are also omitted in this schematic, e.g., other sources of electron absorption in the detector.

The geometry of the detector and chamber influences the signal intensity, and the influence depends on the deflection of the beam. A schematic of this can be seen in Figure 2. The solid angle Ω of the detector decreases geometrically with larger deflection angles, i.e., $\Omega_{|}>\Omega_{\setminus }$, leading to lower signal intensities. The solid angle is also affected by the size of the opening in the heat shield, which obscures more electron trajectories at higher deflections. Furthermore, the angular distribution ω tends to follow the angle of reflection, while Ω typically follows the angle of incidence. Therefore, the fraction of BSEs that hit the detector, i.e., the intersection Ω∩ω, will decrease with increased deflection. Together, these three effects—(i) solid angle Ω of detector, (ii) heat shield obscuration and (iii) angular distribution ω—decrease the signal intensity with increased beam deflection. This phenomenon was recently termed Surface Tilt and Solid Angle (STSA) contrast [3]. STSA-contrast must be corrected for when using the ELO signal quantitatively. It must also be mentioned that the BSEs that scatter into the heat shield have a non-zero probability of being reflected and potentially hitting the detector. ELO measurements with and without a heat shield have shown that the heat shield acts as a funnel, directing electrons into the detector, increasing the ELO signal by roughly 10%.

### 2.6. Topographical Contrast

A flat and polished surface will appear brighter, while a tilted or rough surface will appear darker, since their BSEs tend to scatter at larger angles, deviating from the direction towards the detector. This allows for topographical features such as bulging, porosity and delamination to be distinguished. A less dense specimen will also have a lower brightness due to increased internal scattering. Sintered powder and subsurface cavities are therefore darker than fully consolidated material. Topographical and material contrast are indistinguishable from one another on a single-detector ELO system, unless additional context is available. Worth noting is that topographical features will never increase the BSE signal, although some odd exceptions do exist.

### 2.7. Other Sources of Electrons

BSEs are not the only electrons that exit the surface. Secondary electrons (SE) and Auger electrons (AE), resulting from elastic interactions with the electron shells, are also able to exit the surface. They are essentially indistinguishable from BSEs, although they typically have different energies, due to the elastic or inelastic nature of their origin. BSEs scatter inelastically, preserving most of the energy they had as PEs. By convention, electrons with energies lower than 50 eV are considered SEs. AEs have characteristic energy levels depending on the energy levels of the atomic shells, and typically range from 50 eV to 2 keV. An advanced electron detector is capable of distinguishing BSEs, SEs and AEs from each other using, e.g., bias voltages or semiconductor electronics. Such advanced detectors are difficult to install in an PBF-EB process, due to the hazardous process environment. Only simple detectors are robust enough to work reliably. Thermionic emissions can also be a factor for certain materials. For most materials, the thermionic emissions are significantly lower than the BSE current, but this may not be true if the material has a high melting point and a low work function. It has also been shown that process gas has an impact on the ELO signal [20]. Helium, an inert gas commonly used in PBF-EB to mitigate the unwanted *smoke* phenomenon, can become ionized and act as a charge carrier. As a consequence, the BSE signal measured using ELO is to be regarded as a superposition of all potential sources for electron absorption in the detector. This motivates the addition of *m* in Equation (3).

## 3. Experimental

### 3.1. PBF-EB Machine and Material

The study was conducted on a novel PBF-EB system, named *HELIOS*, located at Neue Materialien Fürth GmbH (Fürth, Germany). The machine is freely programmable and runs on software developed by the Chair of Material Science and Engineering of Metals from the Friedrich-Alexander-Universität in Erlangen-Nürnberg (Germany). The machine was developed and built by pro-beam GmbH & Co. KGaA (Gilching, Germany) and is equipped with a modified electron beam welding gun, capable of 150 kV acceleration voltage and up to 45 kW beam power. A single backscattering detector is mounted at the top of the build chamber, enabling process monitoring with ELO. The 150 kV acceleration voltage is substantially higher than that of other PBF-EB machines available on the market. Importantly, the high acceleration voltage greatly improves the electron optics, thereby improving the ELO imaging capabilities. This makes the imaging capabilities of the *HELIOS* unique in the field of PBF-EB.

**Figure 3:**
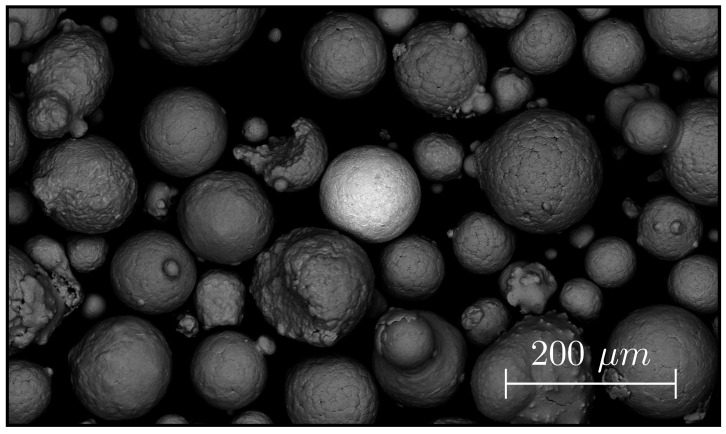
SEM image of TNM powder with a single tantalum-contaminated powder particle. The tantalum increases the backscattering coefficient, making the particle brighter. The tantalum mass concentration was measured to be 27%, via EDXS. The image has been contrast enhanced.

TNM, a third generation titanium aluminide alloy, was used in the study [21]. The powder was produced via gas atomization and possesses the nominal composition Ti-29.4Al-9.1Nb-2.4Mo (wt%). The powder has been reused multiple times over a span of two years and generally shows good processability. At the time of the experiment the powder size was 45–150 μm ($D_{V,50}=103\;\mu m$), determined via laser diffraction by the Mastersizer 3000 (Malvern Instruments Ltd., Worcestershire, UK), and the oxygen content was 0.125 wt%, measured via carrier gas hot extraction with the EMGA 620W (HORIBA, Kyoto, Japan). The powder is spherical with few satellites. In this study, it was discovered that a tiny fraction of the powder particles wrongfully contain large amounts of tantalum. One such particle can be seen in Figure 3.

### 3.2. Inclusion Detection Experiment

The Inclusion Detection Experiment was conducted to detect and preserve inclusions created when building cuboid samples in the contaminated TNM powder. The inclusions were detected digitally using image analysis. Upon detection, all subsequent layers of the cuboid were cancelled, preserving the inclusion on the surface of the sample, encapsulating it in sintered powder. A total of 25 cuboids (15×15 mm) were built for a total of 110 layers. A parameter set known to produce dense samples was used: 150 kV beam acceleration, 6 mA beam current, 100 μm line spacing and 6 m/s scanning velocity with a cross-snake scan strategy. The temperature of the powder surface was held at approximately 1050 °C, measured from the bottom of the start plate. The chamber vacuum was $2\times 10^{-5}$ mbar. ELO images were taken with 2.0 mA beam current, 150 kV acceleration voltage and signal amplification A=20. Approximately 1 μA in the ELO detector corresponds to 0.2 V after amplification. Using the pinhole method, the D4σ has been measured to be 400 μm, and the electron distribution does not significantly deviate from a Gaussian. The images were taken with 1500×1500 pixel resolution, each pixel $80\times 80\;\mu m^{2}$, covering a total area of $120\times 120\;mm^{2}$. Note that the D4σ diameter is five times the pixel length. Each pixel is sampled for 0.5 μs, achieving a signal-to-noise ratio of 30. Specifically, 10 data points are recorded per pixel (0.05 μs per data point) achieving a sampling rate of 20 MHz. After cooling down, the samples were sandblasted to remove the surrounding powder cake and cleaned in an ultrasonic bath. No surface treatments were performed. An FEI Helios NanoLab 600i Scanning Electron Microscope equipped with an Energy Dispersive X-ray Spectrometry (EDXS) detector was used to take SEM and EDXS images. The SEM images were taken with 15 kV acceleration voltage and 0.69 nA beam current. Additionally, a small amount (<1 g) of TNM powder was placed in the SEM and examined manually in search of contaminated powder particles.

### 3.3. Digital Image Analysis of ELO Images

The image analysis script was written in Python to identify the inclusions. The functionality of the script is based on the scikit-image library [22]. The script is publicly available on github.com/MartinGardfjell (accessed on 1 June 2023). The core features are:

- Identifying the contours of the printed surfaces using the Watershed algorithm
- Enhancing feature contrast within the contours using Sobel’s Edge Detection algorithm
- Removing non-enclosed features by flood filling
- Identifying small circular features using Hough’s Circle Transformation

### 3.4. Material Contrast Calibration Plate

**Figure 4:**
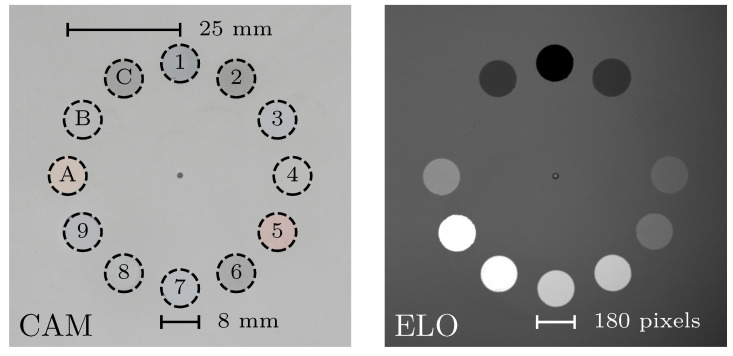
Images of the Material Contrast Calibration Plate taken with a regular camera (CAM) and with Electron Optical imaging (ELO). The materials are numbered according to Table 1. Notice that aluminum (1) has the darkest intensity in the ELO image, and tungsten (9) the brightest, as is to be expected from their atomic numbers. Iron (3) and Stainless Steel (B) are indistinguishable from the stainless steel baseplate.

Signal intensities from known materials are required to calibrate the material contrast in the ELO. Therefore, the Material Contrast Calibration Plate was built, as can be seen in Figure 4. Starting with a standard stainless-steel base plate, twelve 8 mm holes were drilled, in which rods of different materials were pressed. Nine pure elements and three common alloys were investigated, the details of which can be seen in Table 1. The circular positioning design was chosen in an attempt to minimize the difference in STSA-contrast between the samples. The surface was polished by hand before imaging, to minimize topographical contrast. Using 150 kV acceleration voltage, ELO pictures were taken for five different beam currents between 1 mA and 5 mA. The chamber vacuum was $2\times 10^{-5}$ mbar and the temperature ambient. The images were taken with 1500×1500 pixel resolution, with a pixel size of $45\times 45\;\mu m^{2}$, covering a total area of $67.5\times 67.5\;mm^{2}$.

## 4. Results

### 4.1. Inclusion Detection Experiment

**Figure 5:**
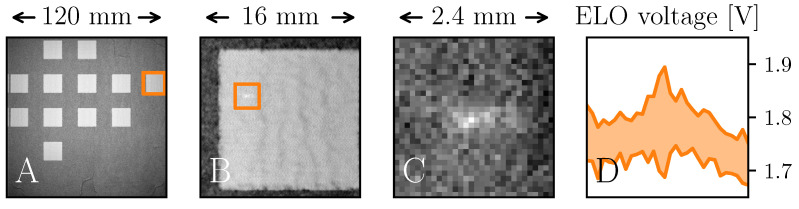
An ELO image from the Inclusion Detection Experiment (**A**), in which the right-most cube contains an inclusion, indicated by the orange square. Zooming in on the cube (**B**), and zooming in once more on the inclusion (**C**), a slightly brighter region appears, originating from the tantalum. Treating (**C**) as a matrix, the maximum and minimum values of its columns are plotted to visualize the signal intensities (**D**). A clear peak can be observed roughly 0.1 V higher than the surrounding TNM.

**Figure 6:**
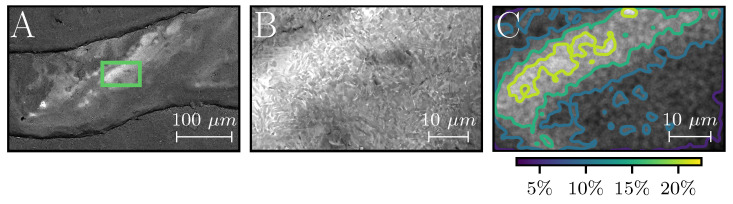
SEM image of a tantalum inclusion on the as-built surface (**A**). At a higher magnification (**B**) the crystal structures can be seen, which is quite exceptional for an as-built surface. The brighter regions contain more tantalum, upwards of 25 wt%. The distribution of tantalum (**C**), was measured with EDXS.

Five inclusions were successfully identified and preserved in the Inclusion Detection Experiment, one of which can be seen in Figure 5A–C. The peak ELO signal intensities of the inclusions reached upwards of 1.9 V after STSA-correction, while the signal from the TNM surrounding the inclusions varied mainly within 1.7–1.8V, as can be seen in Figure 5D. All five inclusions showed strong material contrast in the SEM images, see Figure 6A. The inclusions were all smaller than D4σ, with areas within the range of 0.01 mm^2^ to 0.1 mm^2^, comparable to the area of 2–16 pixels in an ELO image. As a reference, the D4σ area corresponds to roughly 20 pixels. The EDXS measurements determined that the inclusions contained large amounts of tantalum, peak values reaching 25 wt%, as can been seen in Figure 6C. For a pixel-sized region surrounding the centers of the inclusions, the average tantalum concentrations were measured to be around 10–15 wt%, e.g., the elemental composition was measured to be Ti-22.1Al-13.3Ta-6.9Nb-1.6Mo (wt%) for the area seen in Figure 6B, henceforth on referred to as TNM-13Ta. A closer look at Figure 6B reveals a nearly lamellar (NL) microstructure, typical for γ-TiAl processed via PBF-EB [23]. The lamellar features are faint due to the unpolished surface, and therefore grains consisting of $\alpha_{2}$ and γ lamellae appear gray. Between the grains the brighter β-phase can be observed. The β-phase has a higher material contrast, because refractory elements, such as niobium or tantalum, tend to stabilize and segregate into the β-phase in γ-TiAl alloys, due to their partitioning coefficient [24]. This shows that the tantalum was dissolved in the liquid melt pool. The ability to see the crystal structure is a strong indication of good surface quality, and thereby low topographical contrast. No other foreign elements were observed.

A single contaminated powder particle was found in the TNM powder; see Figure 3. Its elemental composition was measured to be Ti-17.1Al-27.1Ta-5.6Nb-0.7Mo (wt%), henceforth referred to as TNM-27Ta. Note that the particle has a spherical surface, which affects the EDXS measurement. No other particles were found despite extensive searching.

### 4.2. Material Contrast Calibration Plate

**Figure 7:**
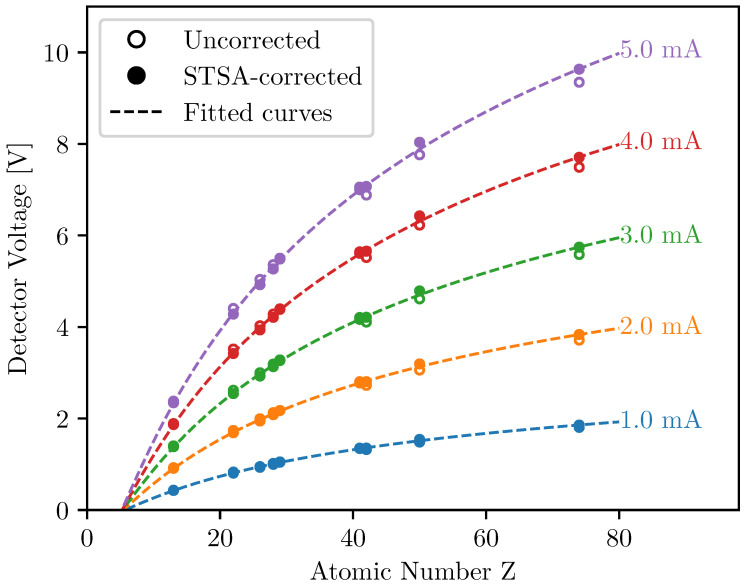
The uncorrected and STSA-corrected values of the pure elements measured on the Material Contrast Calibration Plate are here plotted against their atomic numbers for each of the five different beam currents. Equation (3) has been fitted to the STSA-corrected data, see Table 2.

The pixel intensities of the pure metals on the Material Calibration Contrast Plate are plotted against their atomic number in Figure 7. Fitting the data to Equation (3) yields the values for constants *k*, *l* and *m* found in Table 2. The constants do not differ much between the different beam currents, indicating that the design of the equation is robust. The constants also resemble Equation (2), with l≈9 and m≈0. The mean square error $\epsilon_{Z}\approx 0.556$ shows that the fitting is within roughly one atomic number. $Z^{\mathrm{eff}}$ for alloys A, B and C were calculated using Equations (7), and can be seen in Table 3. The difference $\Delta Z=\bar{Z}-{\bar{Z}}^{\mathrm{eff}}$ is less than half an atomic number for all three alloys, indicating that Equation (7) is capable of calculating an effective atomic number from the elemental composition of an alloy.

### 4.3. In Situ Material Characterization

From the Inclusion Detection Experiment it was determined that the ELO signal spans an interval from 1.7 V to 1.9 V. With the EDSX measurements, the elemental compositions TNM-13Ta and TNM-27Ta were measured, and using Equation (7) their effective atomic numbers could be estimated, see Table 3. TNM-27Ta is the best representation of the composition of the contaminated material. However, such high concentrations of tantalum will never be measurable with the ELO signal, given the small specimen size. Instead, the peak ELO intensity of 1.9 V should correspond to the $Z^{\mathrm{eff}}$ of TNM-13Ta, since this is the average elemental composition of a pixel-sized area.

**Figure 8:**
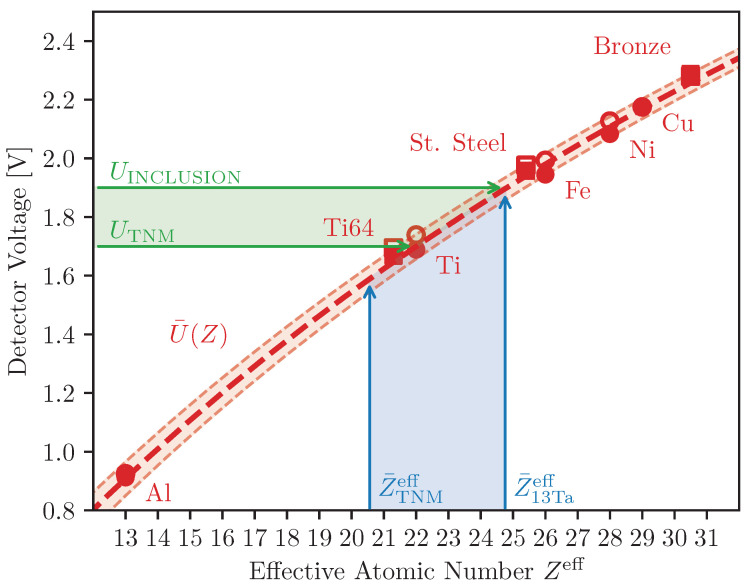
A visual comparison between the voltage measured in the ELO images (green), and the $Z^{\mathrm{eff}}$ calculated from the elemental composition of TNM and TNM-13Ta (blue). The voltages and atomic numbers are comparable through $\bar{U}\left(Z\right)$ (red), which is included with an error margin of ±$\epsilon_{Z}=0.558$. The data points of the pure elements (circles), with (filled) and without (unfilled) STSA-correction. Some alloys are also included (squares). The blue and green regions coincide on $\bar{U}\left(Z\right)$, indicating that in situ material characterization is possible with ELO.

From Figure 5 the ELO signal of TNM, with and without inclusion, is seen to span 1.7–1.9V. Using the Material Contrast Calibration Plate the fitting constants *k*, *l*, and *m* in Equation (3) were determined. With $\bar{U}\left(Z\right)$ established, the effective atomic numbers between TNM and TNM-13Ta can be calculated as the span 20.56–$24.75\;Z^{\mathrm{eff}}$. A comparison between the two spans has been visualized in Figure 8, which shows a rather good fit. $U_{INCLUSION}$ fits almost perfectly with ${\bar{Z}}_{13\mathrm{Ta}}^{\mathrm{eff}}$. The difference of $\bar{Z}\left(1.75\;V\right)-{\bar{Z}}_{TNM}^{\mathrm{eff}}\approx 2.13\;Z^{\mathrm{eff}}$ is however almost four times that of $\epsilon_{Z}$. The comparison shows strong indications that it should be possible to quantitatively determine $Z^{\mathrm{eff}}$ in situ from an ELO intensity with the presented approach.

## 5. Discussion

The initial interpretation of the results has been split into the three sections *Resolution*, *Detection* and *Characterization*.

*Resolution* is the key feature for any imaging tool. Figure 3, showing the SEM image of the 100 μm tantalum contaminated particle, demonstrates the size of the specimen being imaged in this study. A powder particle will become larger when melted, as seen in Figure 6A, but it will still be significantly smaller than the beam. Distinguishing such small specimen is exceptional, especially regarding the fact that a PBF-EB machine is designed for production and not analysis, and even more so when taking into account that the ELO detector is only a simple aluminum plate connected to an amplifier. Understanding how the ELO achieves a resolution smaller than the beam is very important. The resolution depends greatly on the profile of the beam in the powder plane. How the profile is influenced by the machine controllable parameters, e.g., beam current and acceleration voltage, is not well studied. Simulating the relationship between the machine parameters and the beam characteristics could shed light on this topic, as has been attempted previously by Gardfjell [12]. Especially interesting would be to investigate how it is even possible to detect a 100 μm specimen with a D4σ=400 μm beam, when only $1-e^{-\frac{1}{8}}=11.75\%$ of the electrons hit the sample. An important comment is that ELO imaging uses oversampling to ensure that even small specimen are interacting multiple times with the electron beam. The approach of oversampling prevents small objects from being skipped. The pixel size should always be significantly smaller than the beam, and the sampling time significantly long to achieve a good signal-to-noise ratio. Current research also attempts to improve the ELO system by introducing multiple detectors [3]. A multi-detector system provides enhanced capabilities for distinguishing topographical and material information from each other by either adding or subtracting the signals of opposing detectors.

*Detection* entails extracting features quickly and reliably from the high resolution images. It is the process of transforming the raw numerical image data into verbal or graphical insights. Detection is currently only performed manually on the ELO, which is not only time-consuming, but also very subjective, with interpretations depending greatly on the experience and skill of the operator. Automated process monitoring would decrease costs, while greatly improving the amount, and quality, of insights gathered using the ELO. In this study it was shown how process monitoring could be used to detect powder contamination. The tantalum contamination was neither observable with routine powder measurements nor with high resolution XCT scans. Only with high resolution ELO images were the inclusions detectable, and only with digital image analysis were they detected fast enough to be preserved. This shows the importance of automated process observations.

*Characterization* is the process of mapping the ELO signal intensities to corresponding effective atomic numbers. While *detection* only requires relative difference between pixels to distinguish features, *characterization* requires the absolute signal values. This requires a complete understanding of all the influencing factors in the imaging process. The framework presented in Section 1 attempts this and is quite successful at determining the effective atomic numbers of alloys A–C on the Material Contrast Calibration Plate, as is seen in Table 3, where ΔZ is smaller than $\epsilon_{Z}=0.558\;Z^{\mathrm{eff}}$. The framework is also quite successful at determining the effective atomic number in situ. It correctly predicts the effective atomic number of TNM-13Ta, but is less accurate for TNM, where $\Delta Z=2.13\;Z^{\mathrm{eff}}$. These results show that there is potential to use the ELO detector to characterize materials in a PBF-EB process, although more data must be gathered before this process can be verified for any arbitrary material. With that said, it should be repeated that the process can only predict the effective atomic number, never the elemental composition, given that different alloys can have the same $Z^{\mathrm{eff}}$.

It is worth discussing why alloys A–C perform better within the framework than the TNM alloys. Fundamentally, it is a difference of relative vs. absolute data. The images of alloys A–C were gathered simultaneously as the pure elements 1–9, which are used to calibrate the relationship. The TNM images were gathered at a different time under slightly different circumstances, meaning the calibration is not applicable unless the difference in circumstances are corrected for. Two big differences between the experiments are surface quality and specimen size. The Material Contrast Calibration Plate has flatter surface than the as-built samples, which should introduce a small overestimation, i.e., the predicted voltages should be slightly higher then the measured ones. The opposite is however observed in Figure 8, where the measured voltage of TNM is higher than the predicted $Z^{\mathrm{eff}}$. The tantalum inclusions are smaller than the beam and therefore the signal $U_{INCLUSION}$ should be influenced by the surroundings of the inclusion. This is compensated by using TNM-13Ta, and not TNM-27Ta, for the comparison, but it is still surprising how exact the prediction is. Contrarily, $U_{TNM}$ is measuring a sample larger than the beam, and should therefore be expected to fit better than $U_{INCLUSION}$, which it does not. Perhaps there is some minor effect missing in the framework that slightly shifts the predicted $Z^{\mathrm{eff}}$. It is also possible that the elemental composition for TNM has shifted during the melting process.

Another big difference between the measurements is the temperature. The temperature of a TNM build process is typically 1050 °C on the surface of the powder plane. The temperature should not affect the backscattering coefficient, but it could yield significantly high amounts of thermionic emissions from the powder surface. The high temperature also produces evaporation, where lighter elements, such as Al, evaporate at higher degrees, leaving behind a heavier alloy, with a higher backscattering coefficient. A lot of evaporation is, however, required to see a large change in the atomic number. The PBF-EB process typically causes 1 wt% Al evaporation, which for TNM only increases the effective atomic number by approximately 0.1 $Z^{\mathrm{eff}}$. The temperature should also heat up the rest of the machine, including the ELO detector. With a vacuum environment, heat is mainly transferred to the detector through radiation. The temperature of the detector is known to have reached temperatures upwards of 340 °C during very long builds. A temperature gradient between the ELO detector and the reference plate could affect the signal. A temperature gradient between the ELO plate and the amplifier could induce a current, i.e., a thermoelectric force. The heat could perhaps also affect the electrical components in the circuits. Metal vapor condensates on all surfaces of the machine, including the ELO detector, and therefore it is possible that the metallization acts as a charge carrier, similar to process gas. As mentioned previously, 1 μA hitting the ELO detector will be measured as 0.2 V in the amplifier. Whether these effects can induce a μA current, individually or combined, is however difficult to say. Perhaps it is intrinsically foolish to seek a perfect understanding of the ELO signal creation. Instead it could be wiser to focus on a robust calibration procedure that can re-calibrate the ELO signal against known materials before every build job, or perhaps even during.

The mathematical framework should also be scrutinized. The equations used in the framework are based on empirical observations and are not derived from first principles. It can be argued whether there are equations that better describe the relationship, as has been done by R. Herrmann and L. Reimer [19]. Furthermore, it would probably be better to rely less on equations and instead collect more empirical data. The choice of materials on the calibration plate also affects the calibration. In this study, the materials were chosen after price and availability, while achieving a good span of atomic numbers and including all the alloying elements of TNM. In hindsight, most of the materials have atomic numbers between 20 and 30, which probably improves the fitting in this region, while worsening the fitting for heavier materials. Moreover, not all alloying elements were included on the Material Contrast Calibration Plate, and therefore some had to be interpolated, e.g., vanadium in Ti64. Including more materials on the plate would improve the fitting, but it would also make the polishing more difficult, since different materials have different hardness and require different grits.

Characterization is arguably rather difficult to implement and one may ask whether its value is worth its cost. Powder contamination is a very rare occurrence in PBF-EB and companies typically go to great lengths ensuring that their powder handling is controlled. It is probably sufficient to just detect the contamination, without characterization. An application that could benefit from characterization is estimating evaporation rates. In TiAl, aluminum evaporates much faster than titanium, and therefore alloys for PBF-EB are designed to have more aluminum to account for the evaporation. If the process temperature is not kept constant, the behavior of the melt pool can change, which can affect the aluminum concentration in the final part. Temperature is notoriously difficult to measure in a PBF-EB process, and perhaps ELO can be used as a viable alternative.

### Conclusions

- The *HELIOS* demonstrates that it is possible to achieve a detection limit of ∼100 μm in a PBF-EB process with a single-detector ELO system.
- The Inclusion Detection Experiment demonstrates a viable process for preserving and analyzing inclusions, when powder contamination is suspected.
- The Material Contrast Calibration Plate, together with the mathematical framework, presents a novel approach of calibrating the ELO intensity signal to corresponding effective atomic numbers $Z^{\mathrm{eff}}$.
- The characterization of the tantalum inclusions show that there is potential for in situ material characterization with the presented framework, although a lot of work remains to realize it for any arbitrary material.

## Figures and Tables

**Table 1:** Material Specifications.

#	Name	Composition	Z	$\eta {\;}^{**}$
1	Aluminum	99.5 Al	13	0.0660
2	Titanium	99.5 Ti	22	0.1414
3	Iron	99.9 Fe	26	0.1818
4	Nickel	99.6 Ni	28	0.1926
5	Copper	99.99 Cu	29	0.2002
6	Niobium	99.95 Nb	41	0.2814
7	Molybdenum	99.95 Mo	42	0.2906
8	Tin	99.9 Sn	50	0.3160
9	Tungsten	99.95 W	74	0.4336
				
A	Bronze	Cu-8Sn	30.5 *	0.2258
B	Stainless Steel	Fe-18Cr-10Ni-2Mn	25.4 *	0.1772
C	Ti64	Ti-6Al-4V	21.3 *	0.1440

* Effective atomic number estimated using Equation (7). ** Values derived from CASINO simulations.

**Table 2:** Fitting constants and estimated errors.

*I* [mA]	*k*	*l*	*m*	$\epsilon_{U}/I$	$\epsilon_{Z}$
1.0	4.804	10.25	−0.051	0.0107	0.560
2.0	4.896	10.20	−0.048	0.0105	0.543
3.0	4.872	10.17	−0.047	0.0104	0.547
4.0	4.900	10.20	−0.046	0.0106	0.569
5.0	4.891	10.21	−0.045	0.0107	0.571
Average	4.873	10.21	−0.048	0.0106	0.558

**Table 3:** Calculated and measured effective atomic numbers of alloys.

Alloy Γ	U/I	$U_{\Gamma }^{\mathrm{eff}}/I$	$\bar{Z}\left(U\right)$	$\bar{Z}\left(U_{\Gamma }^{\mathrm{eff}}\right)$	$\Delta Z^{\;*}$
Bronze	1.14	1.13	31.13	30.57	0.56
Stainless Steel	0.98	0.97	25.69	25.43	0.26
Ti64	0.83	0.82	21.58	21.29	0.29
					
TNM	∼0.875	0.795	∼22.70	20.56	∼2.13
TNM-13Ta	∼0.950	0.947	∼24.83	24.75	∼0.08
TNM-27Ta	-	1.01	-	29.63	-

* $\Delta Z=\bar{Z}\left(U\right)-\bar{Z}\left(U_{\Gamma }^{\mathrm{eff}}\right)$.

## Data Availability

The data and code are accessible on github.com/MartinGardfjell (accessed on 1 June 2023).
